# Special Issue: “Latest Advances on Urological Surgery”

**DOI:** 10.3390/jcm12134452

**Published:** 2023-07-03

**Authors:** Emilio Sacco

**Affiliations:** 1 Urology at Department of Medicine and Translational Surgery, Università Cattolica del Sacro Cuore, Largo Vito 1, 00168 Rome, Italy; emilio.sacco@gmail.com; 2 Robotic Urology Unit at Urology Department, Fondazione Policlinico Universitario Agostino Gemelli IRCCS, Largo Agostino Gemelli 8, 00168 Rome, Italy

Urology has always been at the forefront of surgical innovation which aims to improve patients outcomes and cost-effectiveness. This Special Issue of the Journal of Clinical Medicine aimed to publish contributions from distinguished authors who actively experience innovations in the field of urological surgery and aim to provide more solid scientific evidence.

Certainly, the most promising field when looking at surgical innovation is robotic surgery with its numerous advantages, including low invasiveness, higher precision, a lower complication rate, and fast postoperative recovery.

Regarding oncological robot-assisted surgery, Gandi C. and colleagues published a study comparing robot-assisted partial nephrectomy to the open technique in the scenario of an off-clamp approach, using propensity score matching and a new pentafecta outcome [1]. No study has compared these two techniques in a purely off-clamp scenario. A significantly shorter operative time, lower blood loss, and a shorter length of hospital stay were observed in the robotic group. Accordingly, the pentafecta was achieved in a significantly higher proportion of patients in the robotic group, confirming the superiority of robotic surgery in the field of partial nephrectomy in terms of important peri-operative outcomes.

The advantages of robot-assisted surgery have also been reported in the field of radical cystectomy; however, published data are still limited. An evaluation of the learning curve of surgical procedures is of outmost importance, particularly for very complex procedures, such as radical cystectomy with intracorporal neobladder. Lombardo R. and colleagues aimed to assess the learning curve of this challenging procedure using a standardized learning curve assessment method (CUSUM method) and multiple outcomes in a single surgeon experience [2]. While 20 cases were found to be sufficient to achieve the plateau, 60 procedures were necessary to benchmark all outcomes defined in the Pasadena international consensus on this procedure. These results are of great interest, although they must be interpreted based on the inherent study limitations adequately addressed by the authors.

Robotic surgery is increasingly adopted to perform procedures which aim to treat benign diseases as well. This is the case of pyeloplasty. The surgical treatment of ureteropelvic junction obstruction has historically been performed with open dismembered pyeloplasty, according to the Anderson–Hynes technique, with a very high success rate. Minimally invasive approaches, and robotic surgery, in particular, offer several advantages. However, published data comparing the robotic versus the open approach are limited. Moretto S. and colleagues published a retrospective comparison between these two approaches evaluating multiple outcomes [3]. While no statistically significant differences were observed regarding the success rate, intraoperative blood loss, the need for postoperative analgesics and antibiotics, and the early postoperative complication rate were significantly lower in the robotic group which was however associated with higher direct costs.

Robot-assisted laparoscopic pyeloplasty is increasingly adopted for hydronephrosis in children as well, and Pakkasjärvi N. and colleagues sought to systematically summarize the published data on pediatric robot-assisted pyeloplasty to decipher the learning process by analyzing learning curves [4]. They underlined several limitations of the available literature in this field and found that competency was inconsistently defined and mostly based on operative time, although the procedure was considered safe during all stages. The observed biases indicate several research needs that can be generalized to the research in the field of the surgical learning process as a whole.

With an increasingly ageing population in western countries, research on surgical outcomes in elderly patients is of great interest. To address the research gap regarding the complications and impact on patients’ quality of life associated with different urinary diversions after radical cystectomy in older patients, Fuschi A. and colleagues compared perioperative outcomes in two groups of patients aged > 75 years with a high comorbidity state who underwent minimally invasive (laparoscopic or robot-assisted approach) radical cystectomy with an intracorporal ileal conduit (Bricker) or single-stoma ureterocutaneostomy [5]. In their retrospective analysis, they found that single-stoma ureterocutaneostomy after radical cystectomy is a valid alternative to ileal conduit, with comparable quality of life and ostomy management 6 months after surgery, reporting fewer perioperative and postoperative complications, a lower operative time, a faster recovery of bowel function, and a lower hospital stay.

In recent years, the surgical treatment of benign prostatic hypertrophy (BPH) has been developed by a multitude of endoscopic and minimally invasive surgical techniques. In particular, to decrease the morbidity associated with the standard approach (the transurethral resection of the prostate or TURP), a variety of lasers and laser-based procedures have been introduced, such as the thulium laser enucleation of the prostate (ThuLEP). Furthermore, several new procedures aim to preserve the ejaculatory function In this context, Trama F. and colleagues [6] performed a prospective observational study to evaluate the efficacy and feasibility of ejaculation-sparing ThuLEP. The key aspect of the proposed technique was the preservation of a small amount of tissue from the lateral lobes at the level of the prostatic apex near the verumontanum in order to preserve the integrity of the ejaculatory muscles. With the technique under investigation, the authors reported a very high rate of ejaculation preservation after 12 months (94.3%).

In the context of BPH laser surgery, Chen S-L and colleagues [7] performed an observational population-based retrospective study evaluating the postoperative bleeding complications among common six laser transurethral techniques and TURP (monopolar or bipolar) using the Taiwan National Health Insurance Research Database. While GreenLight photovaporization (PVP) and thulium laser vaporesection (ThuVARP) of the prostate were associated with a lower incidence of bleeding events and clot retention compared to monopolar TURP, even in patients receiving anticoagulant or antiplatelet therapy, diode laser enucleation (DiLEP) and holmium laser enucleation (HoLEP) did not result in fewer bleeding events than monopolar TURP. By considering several limitations of such a study design, these results suggest that not all laser techniques may be associated to the presumed advantages of this technology, including a lower rate of postoperative bleeding.

In conclusion, this Special Issue provides important new evidence in the field of urological surgery, including both endoscopic and minimally invasive procedures. The included publications represent a very valuable contribution to fill the gaps in knowledge on several aspects of urology. We hope that the results provided by the contributing authors may serve as an important resource for researchers and may encourage future studies.
